# Integrating ISAR and CIRS with ED triage to predict 30-day adverse outcomes in older adults: Development and internal validation of a dual-scale model

**DOI:** 10.1097/MD.0000000000049471

**Published:** 2026-07-03

**Authors:** Cheng Liu, Ying Li, Wei Jiang, Lanxin Ouyang, Di Liu

**Affiliations:** a Emergency Department, The Central Hospital of Wuhan, Tongji Medical College, Huazhong University of Science and Technology, Wuhan, Hubei, China.

**Keywords:** CIRS scale, composite adverse outcome, emergency medicine, ISAR scale, machine learning, older adults, risk prediction

## Abstract

Older emergency department (ED) patients are at significant risk of experiencing composite adverse outcomes such as ED revisits, intensive care unit admissions, and all-cause mortality within a short period. Existing predictive tools primarily utilize single scoring systems, showing limited predictive accuracy and clinical applicability. This study evaluated whether combining the identification of seniors at risk (ISAR) and the cumulative illness rating scale (CIRS) with routinely available ED triage information could provide incremental predictive value for 30-day composite adverse outcomes among older ED patients in China, compared with single-scale and clinical-variable models. A prospective, single-center cohort study was conducted involving older patients aged ≥ 65 years attending a tertiary hospital ED in China from March to May 2024. ISAR and CIRS assessments, along with baseline clinical data, were collected upon ED admission. The primary outcome was a composite adverse event within 30 days, including ED revisit, intensive care unit admission, or death. Variable selection was performed using least absolute shrinkage and selection operator regression. Logistic regression models, including single-scale, combined ISAR + CIRS, and baseline clinical models, were constructed and compared based on discrimination, calibration, and clinical net benefit through decision curve analysis. Model interpretability was further evaluated using analyses. A total of 607 older patients were enrolled, with 216 (35.6%) experiencing composite adverse outcomes within 30 days. The combined ISAR + CIRS model showed the highest area under the curve (AUC) among the compared models (AUC = 0.807; 95% confidence interval [CI]: 0.776–0.839), with a statistically significant improvement over the ISAR-based model (AUC = 0.793; 95% CI: 0.761–0.826). However, the absolute improvement compared with the CIRS-based model was small (AUC = 0.805; 95% CI: 0.773–0.837). The combined model showed favorable calibration and higher net benefit across clinically relevant thresholds. extreme gradient boosting and SHapley Additive exPlanations analyses suggested that ISAR, CIRS, systolic blood pressure, triage level, and pulse rate were important contributors to model output. The combined ISAR + CIRS model provided an interpretable and clinically feasible approach for early risk stratification of older ED patients. Although the improvement in discrimination was modest, integrating acute geriatric risk assessment and chronic comorbidity burden may support more comprehensive risk evaluation alongside routine ED triage and clinical judgment. Further external validation is needed before broader clinical implementation.

## 1. Introduction

The aging global population has significantly increased the demand for emergency department (ED) services, presenting substantial clinical challenges. Elderly patients face elevated risks of short-term adverse outcomes such as ED revisits, intensive care unit (ICU) admissions, and mortality, consuming significant healthcare resources.^[1]^ Rapid and accurate identification of high-risk elderly patients and timely interventions remain critical clinical challenges.

Various tools, including the Identification of Seniors at Risk (ISAR) and the Cumulative Illness Rating Scale (CIRS), are commonly employed for risk assessment in elderly ED patients. While ISAR is widely adopted due to its simplicity, its predictive performance is moderate, with limited specificity and sensitivity for accurately identifying at-risk individuals.^[2,3]^ Conversely, CIRS effectively assesses chronic comorbidities but has limited predictive accuracy when used independently.^[4,5]^

Composite endpoints combining multiple clinically relevant events (e.g., death, ICU admissions, ED revisits) have gained acceptance due to their higher event rates, enhancing sensitivity and clinical relevance compared to single-outcome measures.^[6,7]^ However, most existing research relies on single-scale evaluations or isolated clinical parameters, lacking comprehensive models that integrate ISAR, CIRS, and essential clinical factors, particularly within Chinese populations.^[8,9]^ Additionally, current machine learning applications predominantly prioritize predictive accuracy over interpretability and clinical utility.^[10–13]^ Thus, there is a need to develop comprehensive and interpretable approaches that integrate geriatric vulnerability, comorbidity burden, and routinely available ED clinical information to support early risk stratification. Recent geriatric literature increasingly emphasizes that vulnerability in older adults is multidimensional and cannot be fully captured by a single clinical indicator. Frailty, comorbidity, functional decline, and care needs often coexist and jointly influence adverse outcomes. Fernández-Carnero et al highlighted the need for multidimensional approaches to frailty assessment and the potential role of advanced modeling methods in identifying high-risk older adults.^[14,15]^ This conceptual framework supports the integration of acute geriatric risk assessment and chronic comorbidity burden in ED risk stratification.

This study systematically evaluates a combined ISAR and CIRS model to predict 30-day composite adverse outcomes among elderly ED patients in China, comparing its performance against single-scale and traditional clinical indicators. Additionally, machine learning techniques and SHapley Additive exPlanations (SHAP) analyses are employed to enhance interpretability, addressing current knowledge gaps in risk prediction tools. By integrating ISAR and CIRS, this study aimed to examine whether a dual-scale approach could offer incremental and interpretable value for risk prediction in older adults presenting to the ED.

## 2. Methods

### 2.1. Study design and participants

**Figure 1.**
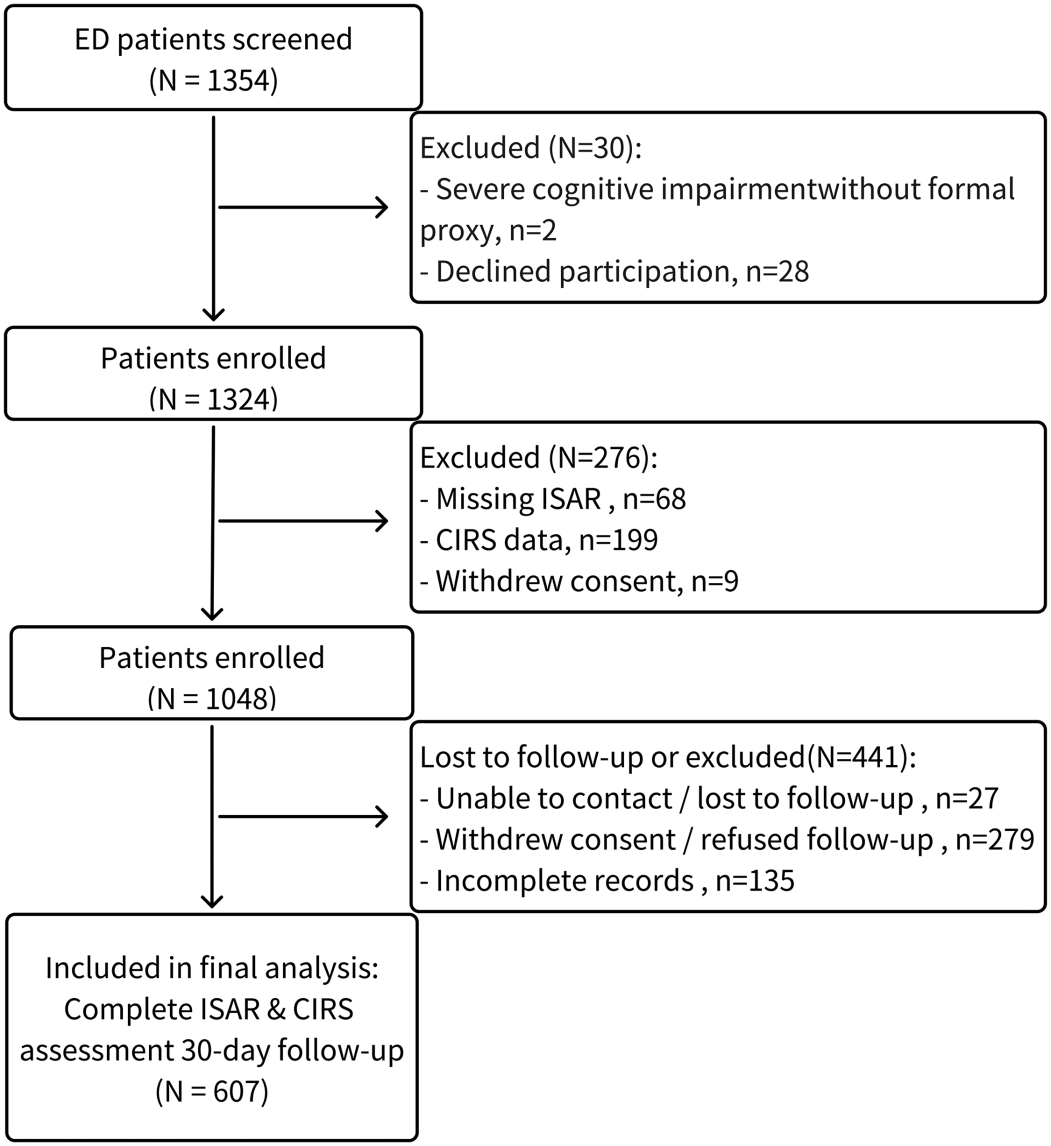
Flowchart illustrating the screening, exclusion, enrollment, and follow-up of older patients included in the study cohort. CIRS = Cumulative Illness Rating Scale, ED = emergency department, ISAR = Identification of Seniors at Risk, N/n = number of patients.

A prospective observational cohort study extended previous elderly ED patient risk assessment projects.^[16]^ The study enrolled elderly patients (≥ 65 years) presenting at a tertiary hospital ED in China from March to May 2024. Patient inclusion and exclusion criteria are detailed in Figure 1. Among 1354 screened older ED patients, 30 were excluded before enrollment. Of the remaining 1324 enrolled patients, 276 were excluded because of missing ISAR assessment, missing CIRS assessment, or withdrawal of consent. Among 1048 patients entering follow-up, 441 were excluded because of loss to follow-up, refusal or withdrawal during follow-up, or incomplete records. Finally, 607 patients with complete ISAR and CIRS assessments and confirmed 30-day outcome data were included in the final analysis. Thirty-day outcomes were primarily ascertained by telephone follow-up. For patients who could not be reached initially, repeated calls were made at different times and on different days. Some older patients could not be contacted because they did not answer unfamiliar telephone calls, blocked unknown numbers, had unavailable telephone numbers, or declined further follow-up. Patients with unconfirmed 30-day outcomes were excluded from the primary analysis because ED revisit, ICU admission, and death required reliable confirmation, and imputation of these outcomes could introduce outcome misclassification. Missing ISAR or CIRS assessments were also not imputed because these scales were core predictors measured at ED presentation, and retrospective reconstruction was considered clinically unreliable. Therefore, the primary analysis was conducted using complete cases. Missingness was not assumed to be completely random, and the potential for selection bias was considered in the interpretation of findings.

### 2.2. Primary variables

Baseline information for all patients was collected by trained emergency care personnel at the time of presentation. This included demographic characteristics (age, sex, residence status, and living support), clinical details (mode of arrival, number of medications, pain score, triage level), and vital signs (temperature, pulse, respiratory rate, systolic blood pressure (SBP), diastolic blood pressure, and oxygen saturation [SpO_2_]). The ISAR is an internationally recognized risk assessment tool for geriatric emergencies. It comprises 6 items: recent hospitalization, visual impairment, cognitive impairment, polypharmacy, limitations in activities of daily living, and need for nursing care. Each item is scored as 0 or 1, with a total score ranging from 0 to 6; higher scores indicate a greater risk of adverse outcomes.^[17]^ The authorized Chinese version of ISAR was used in this study.^[16]^ The CIRS was used to systematically assess the burden of chronic comorbidity. It evaluates 14 organ systems, with each system scored from 0 (no disease) to 4 (extremely severe disease or disability), yielding a total score from 0 to 56. Higher scores reflect a greater comorbidity burden.^[18]^ The original CIRS scoring framework was used in this study.

### 2.3. Outcome measures

The primary outcome was a 30-day composite adverse outcome, defined as the occurrence of any of the following events: admission to an ICU (including emergency, specialty, or comprehensive ICUs) after the index emergency visit; all-cause mortality within 30 days; or return to the ED for any reason within 30 days. The occurrence of any one of these events was considered to meet the primary outcome; otherwise, the outcome was regarded as not having occurred.

### 2.4. Statistical analysis

All statistical analyses were performed using R version 4.4.0 (R Foundation for Statistical Computing). The main R packages included glmnet, caret, pROC, rms, rmda, extreme gradient boosting (XGBoost), and SHAPforxgboost. Continuous variables were summarized as mean ± standard deviation or median with interquartile range, as appropriate. Categorical variables were presented as counts and percentages. Between-group comparisons were performed using the *t*-test or Mann–Whitney *U* test for continuous variables and the chi-square test or Fisher exact test for categorical variables, as appropriate. All statistical tests were 2-sided, and a *P* value < .05 was considered statistically significant. Candidate predictors were screened using least absolute shrinkage and selection operator (LASSO) logistic regression with 10-fold cross-validation. The optimal penalty parameter was selected using the lambda.1-SE criterion to reduce model complexity and improve generalizability. ISAR and CIRS were prespecified as the core predictors of interest and were retained unpenalized in the LASSO procedure. Age was also retained a priori in the final logistic regression model because of its established clinical relevance in geriatric risk assessment, regardless of whether it was selected by LASSO. LASSO was used for predictor screening rather than as the final prediction model. The final prediction models were fitted using multivariable logistic regression because of its transparency, interpretability, and feasibility for clinical implementation. Four models were constructed and compared: a baseline clinical model, an ISAR-based model, a CIRS-based model, and a combined ISAR + CIRS model. Model discrimination was evaluated using receiver operating characteristic (ROC) curves and the area under the curve (AUC). AUCs were compared using the DeLong test. Model calibration was assessed using calibration plots, calibration intercept, calibration slope, and the Brier score. Clinical utility was evaluated using decision curve analysis (DCA) across clinically relevant threshold probabilities. Internal validation of the final combined ISAR + CIRS logistic regression model was performed using bootstrap resampling with 1000 repetitions. Apparent and optimism-corrected performance measures were reported, including AUC and Brier score. Model stability was further evaluated using bootstrap-LASSO resampling across 1000 resampled datasets, and predictor selection frequencies were summarized. The potential risk of overfitting was assessed by calculating the number of outcome events relative to the number of predictors and model parameters, expressed as events per predictor and events per parameter. Because the primary endpoint was a composite outcome consisting of ED revisit, ICU admission, and all-cause mortality within 30 days, exploratory component-specific sensitivity analyses were performed. Using the same predictor set as the final combined ISAR + CIRS model, separate logistic regression models were fitted for 30-day ED revisit, ICU admission, and 30-day all-cause mortality. These analyses were intended to examine whether model performance differed across clinically heterogeneous components of the composite endpoint and were interpreted cautiously because of the smaller number of events for individual outcomes. XGBoost and SHAP analyses were used as supplementary exploratory interpretability analyses rather than as methods for primary model development or internal validation. XGBoost was used to provide an additional nonlinear perspective on variable importance. SHAP were used to visualize the direction and relative contribution of each predictor to the model output. These analyses were intended to support clinical interpretation of the predictor patterns observed in the primary logistic regression framework.

## 3. Results

### 3.1. Baseline characteristics

A total of 1354 older adults presenting to the ED were screened, of whom 607 were included in the final analysis. Baseline characteristics are summarized in Table 1. Overall, 293/607 (48.3%) were male, and the mean age was 76.6 ± 8.1 years. The mean ISAR and CIRS scores were 1.80 ± 1.32 and 6.30 ± 3.33, respectively. When stratified by the 30-day composite adverse outcome (defined as ICU admission, 30-day all-cause mortality, or ED revisit within 30 days) patients with adverse outcomes (n = 216) were older (78.11 vs 75.76 years) and had higher ISAR (2.28 vs 1.54) and higher CIRS scores (7.41 vs 5.68) than those without adverse outcomes (n = 391). Differences were also observed in pulse, SBP, and SpO_2_; triage level and mode of arrival differed between groups as well (details in Table 1).

**Table 1:** Baseline characteristics of older ED patients according to 30-day composite adverse outcome.

	No Adverse Outcome (n = 391)	Adverse Outcome (n = 216)	*P* value
Sex, male, n (%)	194 (49.6)	99 (45.8)	.419
Age, yrs (mean ± SD)	75.76 ± 7.89	78.11 ± 8.04	.001
Living alone, n (%)	21 (5.4)	4 (1.9)	.061
Living support, n (%)	369 (94.4)	202 (93.5)	.805
Mode of arrival, n (%)			< .001
Ambulance	61 (15.6)	94 (43.5)	
Walk-in	207 (52.9)	60 (27.8)	
Assisted arrival	123 (31.5)	62 (28.7)	
ED visit time category, n (%)			.150
8:01–16:00	201 (51.4)	109 (50.5)	
16:01–24:00	152 (38.9)	75 (34.7)	
0:01–8:00	38 (9.7)	32 (14.8)	
Triage level, n (%)			< .001
Level 1	2 (0.5)	13 (6.0)	
Level 2	111 (28.4)	147 (68.1)	
Level 3	177 (45.3)	28 (13.0)	
Level 4	101 (25.8)	28 (13.0)	
Number of medications (mean ± SD)	2.96 ± 2.55	3.52 ± 2.95	.015
Pain score (mean ± SD)	1.18 ± 1.71	0.66 ± 1.34	< .001
Temperature, °C (mean ± SD)	36.52 ± 0.79	36.38 ± 0.64	.028
Pulse, bpm (mean ± SD)	87.43 ± 19.36	94.28 ± 24.71	< .001
SBP, mm Hg (mean ± SD)	147.15 ± 24.23	142.28 ± 29.16	.028
DBP, mm Hg (mean ± SD)	78.30 ± 13.90	77.18 ± 17.51	.386
SpO_2_, % (mean ± SD)	95.95 ± 4.66	93.25 ± 8.59	< .001
ISAR total score (mean ± SD)	1.54 ± 1.24	2.28 ± 1.32	< .001
CIRS total score (mean ± SD)	5.68 ± 2.95	7.41 ± 3.70	< .001

Values are presented as mean ± SD or n (%). “Adverse outcome” refers to 30-day composite adverse outcomes (ICU admission, 30-day all-cause mortality, or 30-day ED revisit).

CIRS = Cumulative Illness Rating Scale, DBP = diastolic blood pressure, ED = emergency department, ICU = intensive care unit, ISAR = Identification of Seniors at Risk, n = number of patients, SBP = systolic blood pressure, SD = standard deviation, SpO_2_ = oxygen saturation.

*P* values were calculated using *t*-test, Mann–Whitney *U* test, or chi-square test as appropriate.

### 3.2. Variable selection and model development

**Figure 2.**
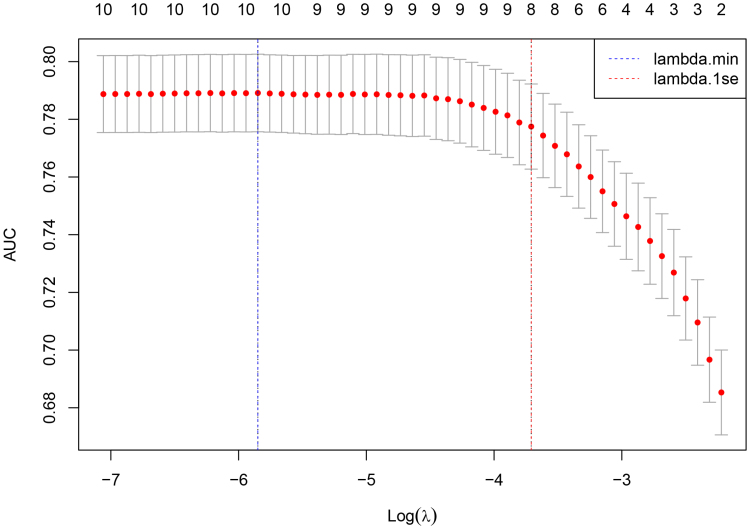
LASSO cross-validation curve showing the selection of the optimal penalty parameter (lambda) and identification of variables included in the predictive model. AUC = area under the curve, LASSO = least absolute shrinkage and selection operator.

LASSO regression was used to screen candidate predictors. Using 10-fold cross-validation and the lambda.1se criterion, 8 nonzero predictors were identified: ISAR score, CIRS score, triage level, pain score, SBP, pulse, temperature, and mode of arrival. The LASSO cross-validation curve is shown in Figure 2.

Because age is a clinically essential demographic predictor in geriatric risk assessment, it was retained a priori in the final logistic regression model regardless of the LASSO selection result. Therefore, the final combined ISAR + CIRS logistic regression model included 9 predictors: ISAR score, CIRS score, age, triage level, pain score, SBP, pulse, temperature, and mode of arrival.

### 3.3. Discriminatory performance of the models

**Figure 3.**
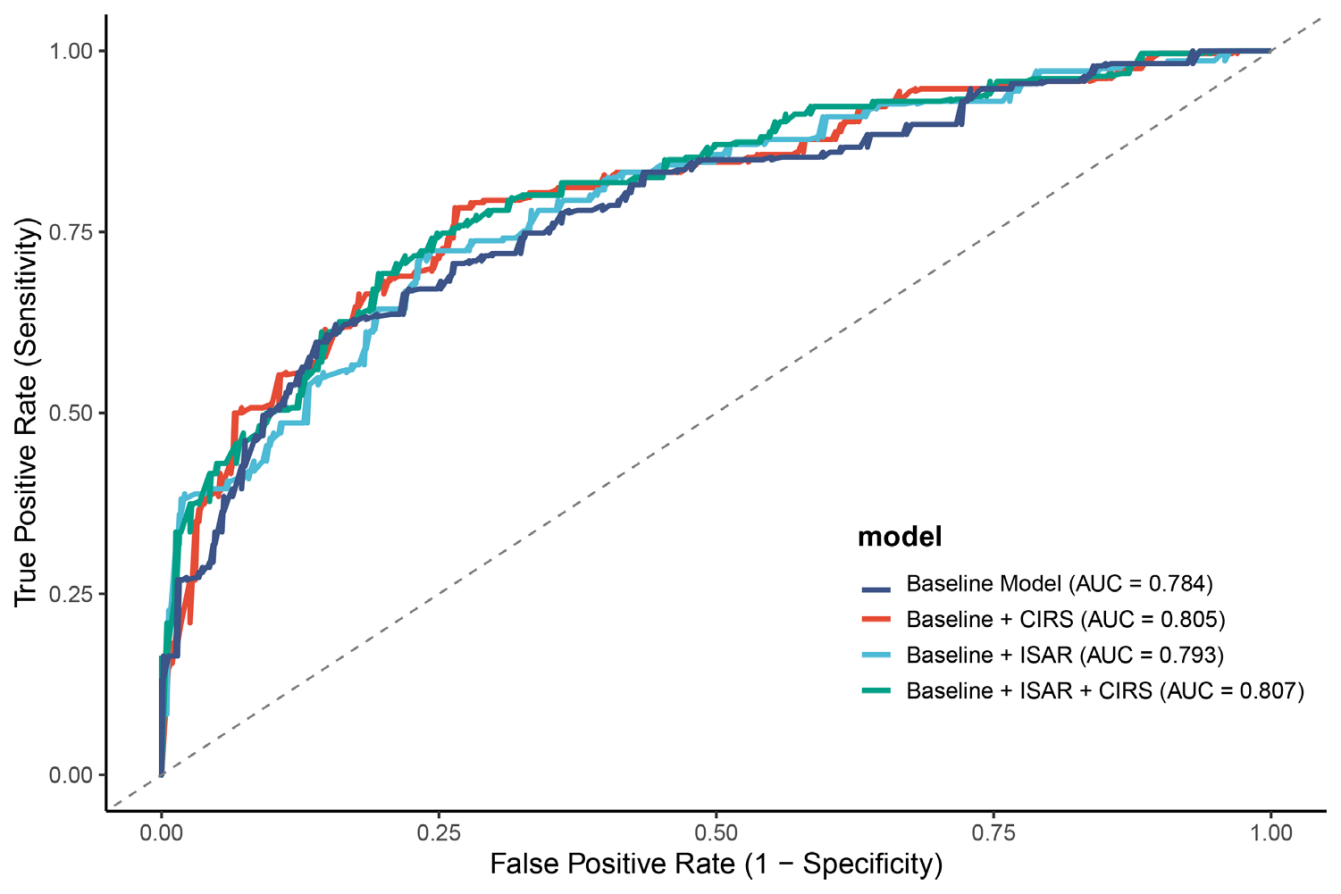
ROC curves comparing the discriminative performance of the baseline clinical model, ISAR-based model, CIRS-based model, and combined ISAR + CIRS model for predicting 30-day composite adverse outcomes in older emergency patients. AUC = area under the curve, CIRS = Cumulative Illness Rating Scale, ISAR = Identification of Seniors at Risk, ROC = receiver operating characteristic.

The discriminative performance of the prediction models was evaluated using ROC curves. Four models were compared: a baseline clinical model, an ISAR-based model, a CIRS-based model, and a combined ISAR + CIRS model (Fig. 3). The combined ISAR + CIRS model achieved the highest AUC among the compared models (AUC = 0.807; 95% confidence interval [CI]: 0.776–0.839). The CIRS-based model showed a similar AUC (AUC = 0.805; 95% CI: 0.773–0.837), followed by the ISAR-based model (AUC = 0.793; 95% CI: 0.761–0.826) and the baseline clinical model (AUC = 0.784; 95% CI: 0.750–0.817). The improvement of the combined model over the ISAR-based model was statistically significant according to the DeLong test (*P* = .002). However, the absolute increase in AUC compared with the CIRS-based model was small. Therefore, the incremental value of the combined model should be interpreted together with calibration and decision curve findings rather than based on discrimination alone. The AUC of 0.807 was derived from the primary ROC comparison among candidate models, whereas bootstrap internal validation of the final combined logistic regression model yielded an apparent AUC of 0.822 and an optimism-corrected AUC of 0.809.

### 3.4. Internal validation, model stability, and overfitting assessment

Internal validation of the final combined ISAR + CIRS logistic regression model was performed using 1000 bootstrap resamples. The apparent AUC was 0.822, and the optimism-corrected AUC was 0.809, indicating modest optimism. The apparent Brier score was 0.155, and the optimism-corrected Brier score was 0.163. The bootstrap calibration intercept was close to 0 (0.000), and the calibration slope was 0.900, suggesting acceptable internal calibration, although mild overfitting could not be completely excluded.

Model stability was further assessed using bootstrap-LASSO resampling. ISAR and CIRS were prespecified core predictors and were retained unpenalized in the LASSO procedure. Among the remaining candidate predictors, triage level showed the highest selection frequency (100.0%), followed by pulse (95.5%), SBP (81.6%), temperature (81.2%), pain score (78.3%), and mode of arrival (66.3%). In contrast, SpO_2_ (46.6%), age (42.7%), and number of medications (6.4%) showed lower selection frequencies.

The final analytic cohort included 216 outcome events. The final model included 9 predictors and 12 model parameters, corresponding to 24.0 events per predictor and 18.0 events per parameter. These findings suggested that the number of outcome events was acceptable relative to model complexity.

### 3.5. Component-specific sensitivity analyses

Exploratory component-specific sensitivity analyses were performed to assess model performance for each component of the composite outcome. For the 30-day ED revisit, 92 events occurred among 607 patients, and the AUC was 0.679 (95% CI: 0.614–0.744). For ICU admission, 120 events occurred, and the AUC was 0.917 (95% CI: 0.895–0.940). For 30-day all-cause mortality, 35 events occurred, and the AUC was 0.862 (95% CI: 0.794–0.929).

These findings indicated that the combined model showed better discrimination for ICU admission and 30-day mortality than for ED revisit. Because the number of events for individual components, particularly mortality, was smaller than that for the composite outcome, these analyses were considered exploratory.

### 3.6. Calibration of the combined model

**Figure 4.**
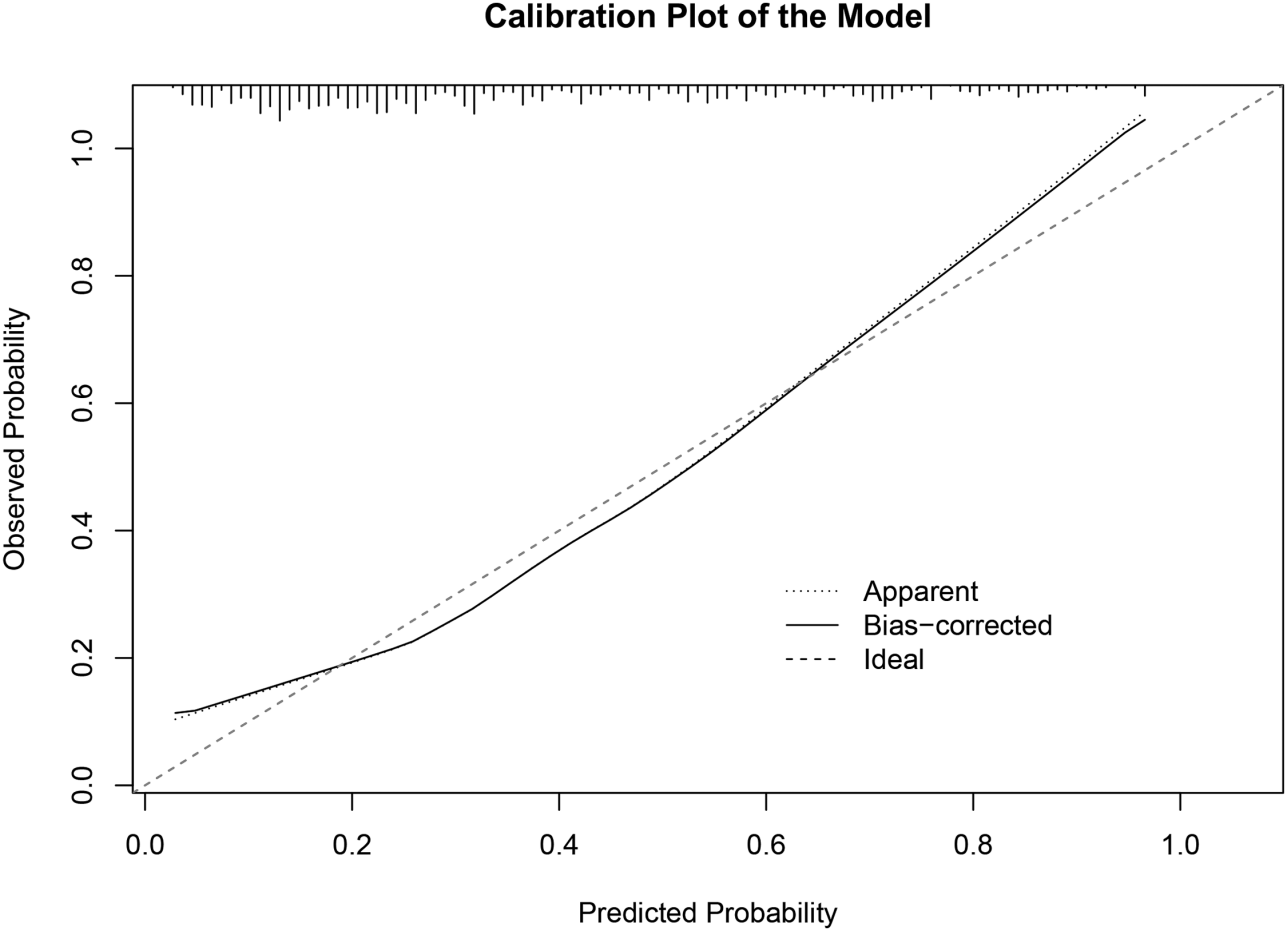
Calibration plot for the combined ISAR + CIRS model, demonstrating agreement between predicted probabilities and observed incidence of 30-day composite adverse outcomes. CIRS = Cumulative Illness Rating Scale, ISAR = Identification of Seniors at Risk.

Calibration of the combined ISAR + CIRS model was assessed using calibration plots (Fig. 4). The calibration curve showed generally acceptable agreement between predicted probabilities and observed event rates. Together with the bootstrap calibration intercept of 0.000 and calibration slope of 0.900, these findings suggested acceptable calibration of the final model, although mild optimism could not be completely excluded.

### 3.7. Clinical decision value of the model

**Figure 5.**
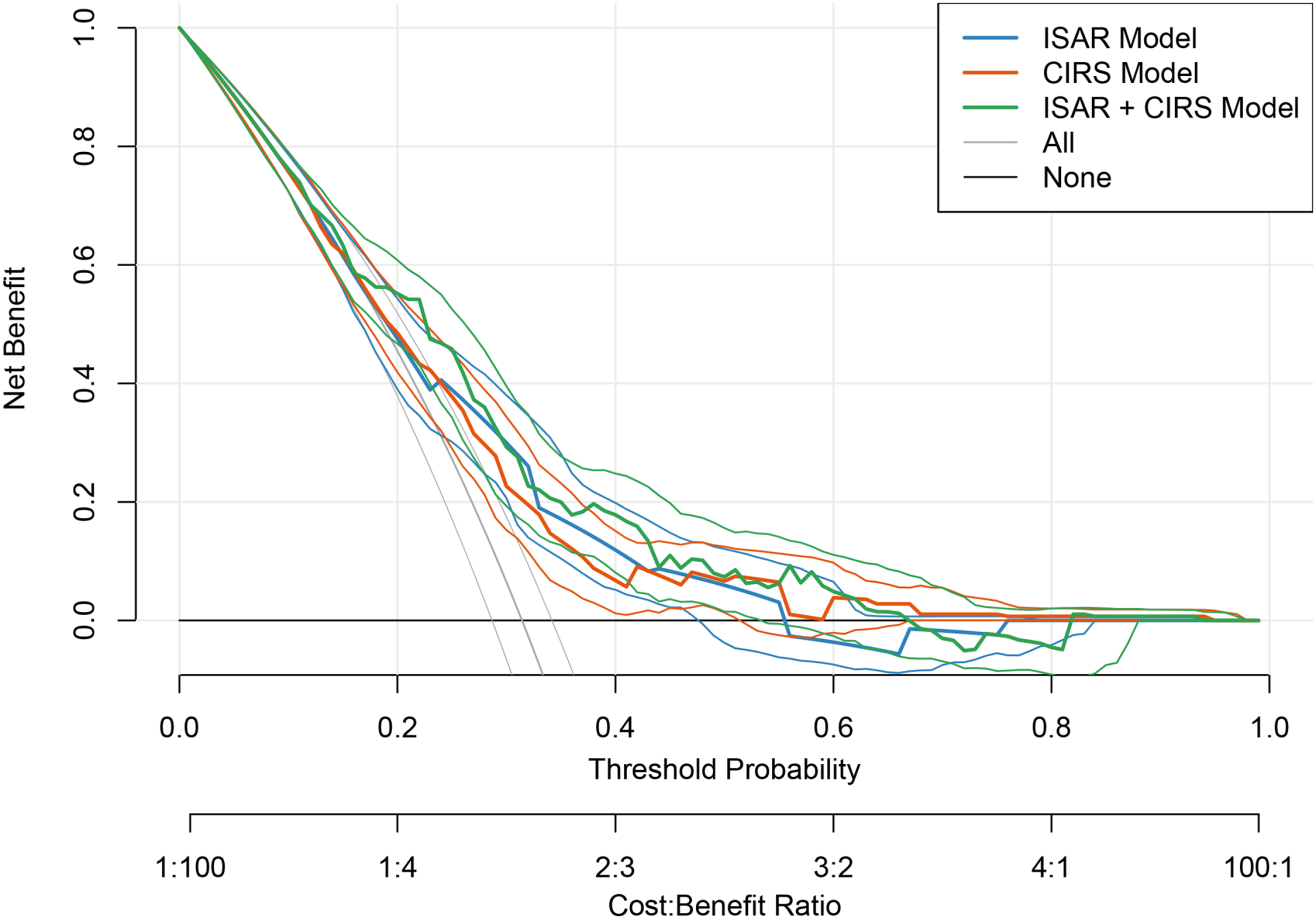
DCA comparing the net clinical benefit of the baseline clinical model, ISAR-based model, CIRS-based model, and combined ISAR + CIRS model across a range of risk threshold probabilities. CIRS = Cumulative Illness Rating Scale, DCA = decision curve analysis, ISAR = Identification of Seniors at Risk.

DCA was used to evaluate the potential clinical utility of the prediction models (Fig. 5). The combined ISAR + CIRS model showed higher net benefit than either scale alone across an approximately 10–40% threshold-probability range. This threshold range may be clinically relevant in ED practice, where clinicians may consider enhanced monitoring, geriatric consultation, careful disposition planning, or early post-discharge follow-up for older patients at moderate predicted risk. However, DCA indicates potential clinical utility under specified threshold assumptions and does not by itself demonstrate improved patient outcomes.

### 3.8. Supplementary XGBoost and SHAP interpretability analyses

As supplementary exploratory interpretability analyses, XGBoost feature importance and SHAP analyses were performed to examine the relative contribution and direction of influence of candidate predictors. These analyses were not used to define or validate the final prediction model, but to support clinical interpretation of the predictor patterns observed in the primary logistic regression framework.

**Figure 6.**
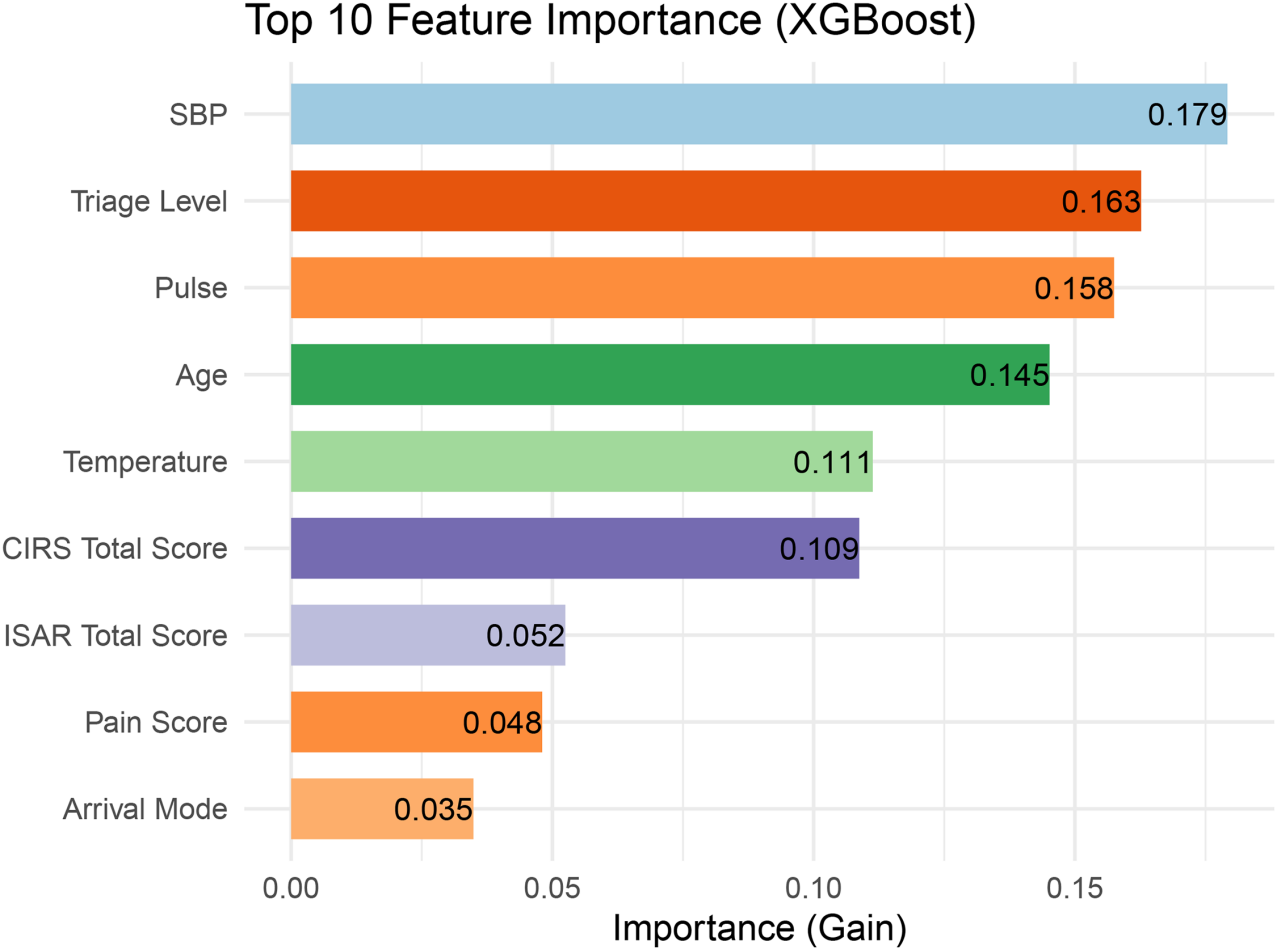
Feature importance ranking derived from the XGBoost model for predicting 30-day composite adverse outcomes, showing the relative contribution of each variable. CIRS = Cumulative Illness Rating Scale, ISAR = Identification of Seniors at Risk, SBP = systolic blood pressure, XGBoost = extreme gradient boosting.

**Figure 7.**
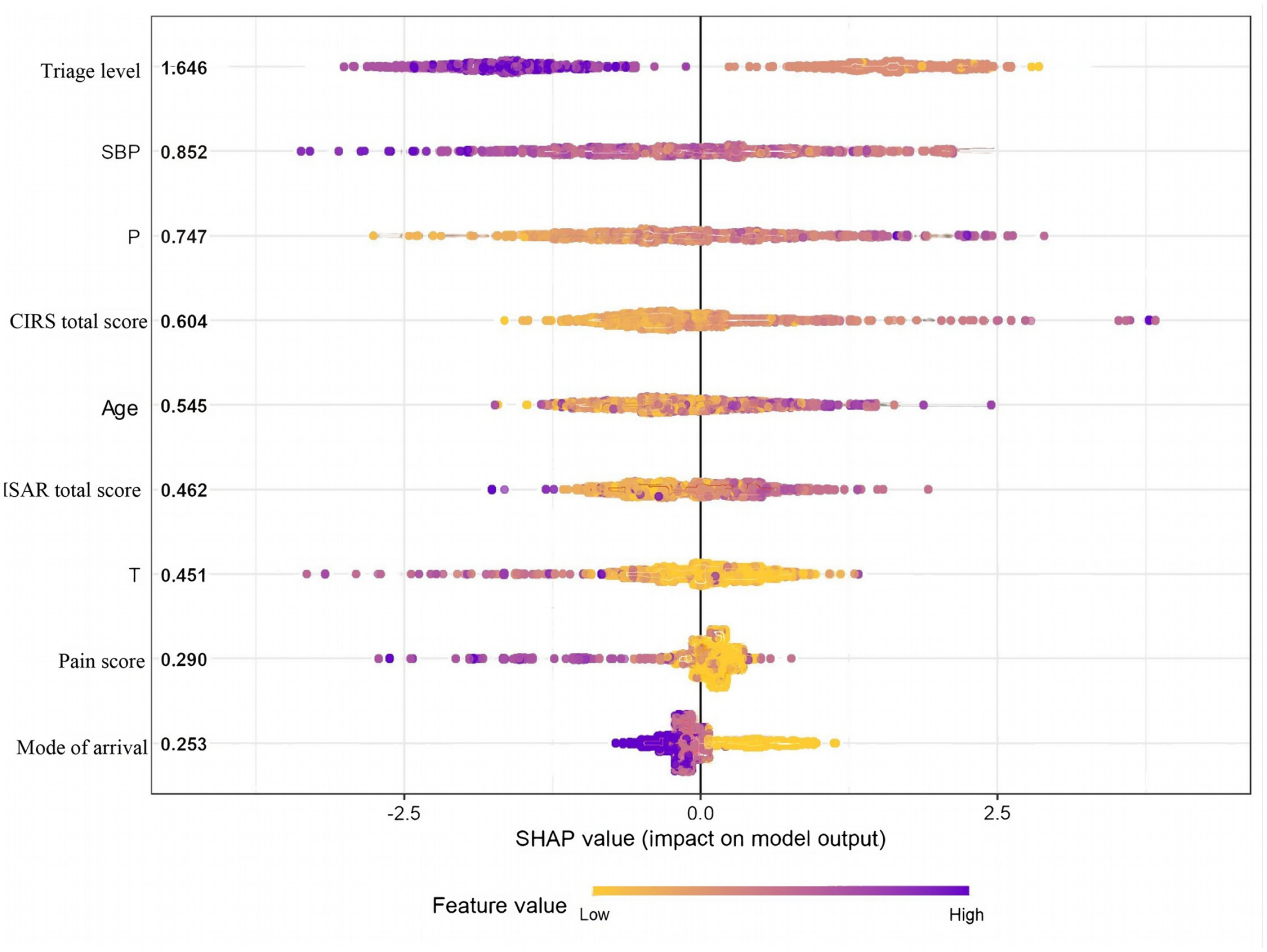
SHAP summary plot illustrating the impact of key features on model output for predicting 30-day composite adverse outcomes among older emergency patients. CIRS = Cumulative Illness Rating Scale, ISAR = Identification of Seniors at Risk, P = pulse, SBP = systolic blood pressure, SHAP = SHapley Additive exPlanations, T = temperature.

XGBoost feature importance analysis identified SBP, triage level, pulse, age, and temperature as the most influential predictors (Fig. 6). CIRS and ISAR total scores also showed meaningful contributions, whereas pain score and mode of arrival contributed less. SHAP analysis further suggested that triage level, SBP, pulse, CIRS total score, and age were important contributors to model output (Fig. 7). The SHAP summary plot indicated that higher triage acuity, higher CIRS and ISAR scores, lower SBP, and higher pulse rates were generally associated with increased predicted risk.

## 4. Discussion

This prospective cohort study evaluated the predictive value of ISAR, CIRS, and their combined use for 30-day composite adverse outcomes among older ED patients in a single-center, real-world setting in China. The combined ISAR + CIRS model achieved the highest AUC among the compared models and showed favorable calibration and decision curve performance. However, the absolute improvement in discrimination was modest, particularly compared with the CIRS-only model. Therefore, these findings should be interpreted as evidence of incremental and clinically interpretable improvement rather than as evidence of a major shift in risk prediction performance.

The rationale for combining ISAR and CIRS is supported by the multidimensional nature of vulnerability in older adults. Older ED patients frequently present with overlapping acute illness, functional vulnerability, chronic comorbidity, polypharmacy, and care needs. Recent geriatric literature has emphasized that frailty and vulnerability in older adults cannot be fully captured by a single clinical indicator and that multidimensional assessment and advanced analytical approaches may improve risk identification in older populations.^[14,15]^ In this context, ISAR and CIRS provide complementary information: ISAR captures acute geriatric vulnerability, functional decline, recent healthcare use, polypharmacy, and care needs, whereas CIRS reflects chronic comorbidity burden across multiple organ systems. Emerging geroscience literature also suggests that aging-related vulnerability may involve interacting clinical, functional, inflammatory, and biological pathways.^[19]^ Although the present study did not include biological markers, this broader framework supports the concept that risk stratification in older adults should integrate multiple dimensions of vulnerability rather than rely on a single score alone.

The predictive performance of the combined model should be interpreted cautiously. Prior research has shown that ISAR alone has only moderate predictive performance for adverse outcomes among older ED patients. For example, Yao et al reported limited accuracy of ISAR in predicting adverse outcomes in older patients seen in the ED.^[3]^ This is consistent with previous evidence suggesting that ISAR is useful as a brief geriatric screening tool but may have limited specificity when used alone.^[2,3]^ Conversely, CIRS provides a structured measure of chronic comorbidity burden, which is clinically relevant in older adults but may not fully capture acute functional vulnerability or ED-specific risk. Previous studies have also shown that comorbidity burden is associated with adverse outcomes and risk stratification in older populations.^[20–23]^ In the present study, the combined ISAR + CIRS model achieved an AUC of 0.807, but this was only slightly higher than the CIRS-only model. Thus, the main contribution of the combined approach may not be a large improvement in discrimination, but rather the integration of complementary clinical domains into a more interpretable risk profile.

The findings are also consistent with the broader modeling literature, suggesting that combining different sources of information may improve prediction compared with relying on a single domain alone.^[21]^ However, the magnitude of improvement in the present study was modest, and this should be acknowledged when considering potential clinical application. The combined ISAR + CIRS model should therefore be viewed as an adjunct to routine ED triage and clinical judgment, rather than as a replacement for existing decision-making processes. Its potential value lies in helping clinicians synthesize acute geriatric vulnerability, chronic comorbidity burden, triage acuity, and vital signs during early ED assessment.

Calibration is an important aspect of prediction model evaluation because discrimination alone does not ensure clinically reliable risk estimates. Van Calster et al emphasized that poorly calibrated prediction models may mislead clinical decision-making even when the AUC appears acceptable.^[24]^ In the present study, the calibration curve suggested generally acceptable agreement between predicted and observed risks. Bootstrap internal validation further showed an optimism-corrected AUC of 0.809 and a calibration slope of 0.900, indicating acceptable internal performance with mild optimism. These results support the internal validity of the combined model, although mild overfitting cannot be completely excluded. This is particularly important because models developed in a single-center cohort may perform differently when applied to other hospitals, populations, or triage systems.

DCA provided additional insight into the potential clinical utility of the model. DCA has increasingly been used to evaluate whether a prediction model may provide net benefit across clinically meaningful threshold probabilities.^[25]^ In this study, the combined model showed higher net benefit than either scale alone across an approximately 10 to 40% threshold-probability range. This threshold range may be relevant in ED practice, where clinicians may consider enhanced monitoring, geriatric consultation, careful disposition planning, medication review, or early post-discharge follow-up for older patients at moderate predicted risk. However, DCA reflects potential net benefit under specified threshold assumptions and does not by itself prove that implementation of the model improves patient outcomes. Therefore, prospective implementation studies are needed to determine whether model-guided decisions can improve care processes, resource allocation, or clinical outcomes in older ED patients.

A particular strength of this study is its emphasis on interpretability. XGBoost and SHAP analyses were used as supplementary exploratory interpretability analyses rather than as the primary model development or validation strategy. The primary prediction framework was based on LASSO-assisted predictor screening followed by multivariable logistic regression, whereas XGBoost and SHAP provided an additional perspective on variable importance and directionality. Previous studies have emphasized the importance of interpretability when machine learning approaches are applied to clinical decision support, because transparent and clinically understandable models are more likely to be trusted and implemented in practice.^[26–28]^ In the present study, the machine learning interpretability results identified clinically plausible contributors, including triage level, SBP, pulse, CIRS score, and ISAR score. These findings are consistent with the clinical understanding that acute physiological instability, triage acuity, geriatric vulnerability, and comorbidity burden are relevant to short-term adverse outcomes in older ED patients. Nevertheless, XGBoost and SHAP analyses do not establish causality and should not be interpreted as independent validation of the final logistic regression model.

The use of a composite outcome also requires cautious interpretation. Composite endpoints are commonly used because they increase event rates and may capture a broader range of clinically relevant adverse outcomes.^[6,7]^ However, ED revisit, ICU admission, and all-cause mortality differ substantially in severity and clinical implications. Mortality represents the most severe endpoint, ICU admission reflects acute clinical deterioration or the need for high-intensity care, whereas ED revisit may also be influenced by symptom recurrence, post-discharge support, healthcare accessibility, and patient behavior. In the exploratory component-specific sensitivity analyses, the combined model showed higher discrimination for ICU admission and 30-day mortality than for ED revisit. This suggests that the model may better capture severe clinical deterioration than return visits, which are likely influenced by broader social and healthcare-system factors. Therefore, the composite endpoint should be interpreted as an overall indicator of short-term vulnerability rather than as a homogeneous clinical event.

Several methodological limitations should be acknowledged. First, this was a single-center study conducted in a tertiary ED in China. The findings may be influenced by local patient characteristics, triage workflows, healthcare resources, and clinical practice patterns. Although bootstrap internal validation was performed, no external validation cohort was available. External validation in independent multicenter cohorts is required before broader clinical implementation can be recommended. Second, a complete-case analysis was used because unconfirmed 30-day outcomes and missing ISAR/CIRS assessments were not imputed. This approach was chosen to preserve the validity of outcome classification and core predictor assessment, but it may have introduced selection bias. Missingness was not assumed to be completely random. In particular, patients who were difficult to contact during telephone follow-up may have differed from included patients in social support, communication accessibility, health status, or disease severity. Third, although the number of outcome events was acceptable relative to model complexity, overfitting cannot be fully excluded. The bootstrap calibration slope of 0.900 suggested mild optimism. We attempted to reduce this risk by using LASSO-based predictor screening, limiting model complexity, and performing bootstrap internal validation. Fourth, the component-specific analyses were exploratory because the number of events for individual outcomes, particularly mortality, was smaller than that for the composite endpoint. Finally, XGBoost and SHAP analyses were used only as supplementary interpretability tools and should not be interpreted as causal analyses or independent validation.

Despite these limitations, this study provides evidence that integrating acute geriatric risk assessment with chronic comorbidity burden may support early risk stratification among older ED patients. The combined ISAR + CIRS model is based on clinically interpretable variables that can be obtained during early ED assessment. Its potential value lies in supporting a more comprehensive and structured evaluation of short-term vulnerability, especially when used together with routine triage information, vital signs, and clinical judgment. Further multicenter external validation and prospective implementation studies are needed to confirm its transportability, evaluate its impact on clinical workflow, and determine whether model-guided interventions can improve outcomes for older adults in emergency care.

## 5. Conclusion

The combined ISAR + CIRS model showed favorable discrimination, calibration, and decision curve performance for predicting 30-day composite adverse outcomes in older ED patients. Although the incremental improvement in discrimination over single-scale models was modest, the model may provide an interpretable and clinically feasible adjunct to routine ED triage and clinical judgment. Further external validation and prospective implementation studies are needed before routine clinical use can be recommended.

## Acknowledgments

The authors would like to thank the emergency physicians, residents, and nurses at The Central Hospital of Wuhan for their dedication and care provided to all patients included in this study.

## Author contributions

**Conceptualization:** Cheng Liu, Ying Li, Di Liu.

**Data curation:** Cheng Liu, Lanxin Ouyang, Di Liu.

**Investigation:** Cheng Liu, Wei Jiang, Di Liu.

**Methodology:** Cheng Liu, Ying Li, Wei Jiang, Lanxin Ouyang, Di Liu.

**Project administration:** Cheng Liu, Di Liu.

**Resources:** Cheng Liu, Wei Jiang, Lanxin Ouyang, Di Liu.

**Software:** Ying Li.

**Supervision:** Wei Jiang.

**Writing – original draft:** Cheng Liu, Ying Li.

**Writing – review & editing:** Di Liu.
